# Additional evidence that the rat renal interstitium contracts *in vivo*

**DOI:** 10.1371/journal.pone.0225640

**Published:** 2019-11-27

**Authors:** Manuel Rodríguez-Martínez, Juan Francisco López-Rodríguez, Omar Flores-Sandoval, Miriam Zarahí Calvo-Turrubiartes, María Eugenia Sánchez-Briones, Ana Sonia Silva-Ramírez, Vianney Guerreo-Ojeda

**Affiliations:** Integrative Physiology Laboratory, Department of Physiology & Biophysics, Faculty of Medicine, Autonomous University of San Luis Potosí, San Luis Potosí, México; Max Delbruck Centrum fur Molekulare Medizin Berlin Buch, GERMANY

## Abstract

We recently provided highly suggestive preliminary evidence that the renal interstitium contracts reactively *in vivo*. We demonstrated that renal medullary direct interstitial volume expansion (rmDIVE = 100 μl bolus infusion of 0.9% saline (SS)/30 s) brought about a biphasic renal interstitial hydrostatic pressure (RIHP) response which was abolished when dibutyryl-cAMP was concomitant and interstitially infused. To assess more deeply the feasibility of the concept that the renal interstitium contracts *in vivo*, two experimental series (S1, S2) were performed in hydropenic rats subjected to acute left renal-denervation, hormonal clamping, and control of renal arterial pressure. In S1, RIHP and renal outer medullary blood flow (RoMBF) were continuously measured before and after a sudden micro-bolus (5μl) injection, into the renal medullary interstitium, of SS containing α-trinositol (α-TNS, anti-inflammatory drug) to either two doses 2 or 4 mM (SS + 2 α-TNS and SS + 4 α-TNS groups). No overall differences between groups in either ΔRIHP or %ΔRoMBF time courses were found; however, in the SS + 2 α-TNS group the data were less scattered and the ΔRIHP time course tended to peak faster and then persisted there, so that, this α-TNS dose was selected for S2. In S2, RIHP and RoMBF were similarly measured in rats randomly assigned to three groups: the CTR group (sham time-control), SS group (SS alone), and SS + α-TNS group. The micro-bolus injection of SS alone (SS group) was unable to increase ΔRIHP. The group with no micro-bolus injection (CTR group) experienced a decrease in ΔRIHP. The micro-bolus injection of SS + 2 α-TNS was accompanied by a differential increase in ΔRIHP (vs. CTR and SS groups). These responses were not associated with differential changes among groups in %ΔRoMBF or hemodilution parameters. These results provide additional evidence that the renal interstitium contracts *in vivo*.

## Introduction

Given the *sui-generis* architecture of the renal interstitium [1] and supported both in the well-known phenomenon of cellular subcutaneous tissue contraction [2] and in the fact that renal medullary interstitial cells in culture contract in response to endothelin and arginine vasopressin [3], we recently provided [4] highly suggestive preliminary evidence that the renal interstitium contracts reactively *in vivo*. We reported that, in hydropenic rats subjected to left renal denervation, hormonal clamping, and renal arterial pressure control, the renal medullary Direct Interstitial Volume Expansion (**rmDIVE**) induced by a 0.9% saline bolus infusion (100 μL / 30 s) into the left renal medullary interstitium brought about a biphasic renal interstitial hydrostatic pressure (RIHP) response (early: ~1 mmHg and late: ~ 4 mmHg). When 5mM dibutyryl-cAMP (db-cAMP) was concomitant and interstitially infused, this response was abolished and was not accompanied by any differential changes among groups in the time course of the haematocrit, serum protein concentration, or renal outer medullary blood flow [4]

Because above paradigm was preliminary supported by the fact that db-cAMP abolished a reactive RIHP response induced by rmDIVE, we aimed to submit this paradigm to much more exacting test in order to support the feasibility of the concept. We sought to ascertain whether the micro-bolus (5 μL) injection, into the renal medullary interstitium, of 0.9% saline solution (to minimize the possibility of inducing rmDIVE) containing 2 mM of α-trinositol (D-myo-inositol-1, 2, 6-triphosphate, Ins(1,2,6)P3, α-TNS) was able to induce a differential increase in RIHP compared to either a sham injection or a real micro-bolus injection of solely 0.9% saline solution. Alfa trinositol is a member of the phosphoinositide family with well-proven anti-inflammatory effects in several models of inflammation, where it significantly and markedly reduces both the typical increased negativity of interstitial pressure [5, 6, 7] and the albumin extravasation [5], and therefore has an edema reducing effect. While this drug´s intimate mechanism of action is unknown, all the evidence points to the fact that it results from the centripetal stretching of the fibrillary collagen network that surrounds the extracellular matrix (prone to swelling due to its hyaluronic acid content) as a consequence of an α_2_β_1_ integrin-mediated fibroblast contraction [6].

## Material and methods

### Animals

Two independent series of experiments (S1 and S2) were carried out in adult male Wistar rats derived from the Charles River 003 strain colony. Their initial body weights were between 250 and 275 g and they were housed individually at the animal care facility under a 12–12 light-dark cycle, and temperature and humidity control with free access to a normal sodium rat diet (Rodent diet, Teklad 2018S, Harlan, Maddison, USA) and filtered water. The rats were weighed daily for the 10 days leading up to the experimental day. Only those rats that presents an appropriate body weight gain were admitted into the study, which was approved by the Internal Committee for the Care and Use of Laboratory Animals of the Faculty of Medicine (UASLP). Moreover, the present study was conducted in accordance with the Mexican Guidelines for the Care and Use of Experimental Animals (NOM-062-Z00-1999) which are in line with the Guide for the Care and Use of Laboratory Animals of the National Institutes of Health.

### Surgery

For both series, the rats were anaesthetized with sodium pentobarbital (50 mg Kg‒1, i.p., Barbithal NRV, Holland de Mexico, Morelos, México) followed by an i.m. administration of atropine (0.05 mg Kg‒1, Pisa, Guadalajara, Mexico). Once deeply anesthetized, the rats were placed on a thermostatically controlled table, to maintain their body temperature at 37°C, and mechanically ventilated (Rodent Ventilator, Model E683, Harvard Apparatus, Holliston, MA, USA) at 60 bpm with a mix of atmospheric air and O_2_ via an endotracheal cannula (PE-200). They were also fitted with the following: (a) a double-port PE-50 right femoral vein catheter for infusing solutions and administrating i.v. anaesthesia during the surgery; (b) a heparinized PE-50 left carotid artery catheter for blood sampling; (c) a heparinized PE-50 left femoral artery catheter, which was introduced up to the common iliac artery for the recording of renal arterial pressure (RAP); (d) Roeder knots in superior mesenteric and celiac arteries; (e) a homemade hydraulic aortic occluder placed around the descending aorta above the right renal artery, for controlling RAP; and, finally (f) a bladder catheter. The right kidney was then vascularly excluded by right renal artery ligation in order to ensure the adequate perfusion of the left kidney, which underwent surgical and chemical (10% phenol/absolute alcohol) acute renal denervation and the destruction of all visible left perihilar lymphatics. The left kidney was then placed dorsal side up into a holder that allowed respiratory movement isolation and avoided ureteral and vascular compression. Once positioned, a custom-made 6 mm-long stainless steel interstitial catheter (31G gauge, unbevelled, 90° angled-tip), connected by a PE-10 catheter to a 10 μl Hamilton syringe (#801, Hamilton Co, Reno Nevada, USA) was implanted into the lower quarter of the greater curvature of the kidney, so that its tip came up to the boundary between the inner strip of the outer medulla and renal papilla. In Series 1, this catheter was purged with either SS + 2 mM of α-trinositol (SS + 2 α-TNS group) or SS + 4 mM of α-trinositol (SS + 4 α-TNS group), while, in Series 2, this catheter was purged with solely SS in Group 1 (CTR group) and Group 2 (SS group), while, in Group 3, the catheter was purged with SS + 2 mM of α-trinositol (SS + α-TNS group). Once the catheter was in place, the disposition of a micro-drop of cyanoacrylate between the catheter’s stabilizer and the kidney capsule avoided leaks. A needle (θ = 0.45 mm, 0.15 mm fiber separation) Laser-Doppler probe (Probe 411, Perimed, Järfälla, Sweden) was also inserted into the outer medulla, at an incident angle of 52° and 5.5 mm beneath the surface using a micromanipulator (Model M3301R, WPI, Sarasota, FL, USA) for recording renal outer medullary blood flow (RoMBF). Once in place, a fat pad placed at the probe insertion point sealed the renal capsule. Finally, a homemade, heparinized, unbevelled, PE-10 subcapsular catheter was implanted to measure RIHP [4, 8], and once in place, a micro-drop of cyanoacrylate was deposited between the catheter and the kidney capsule to avoid leaks. To be included in the analysis, an experiment had to show, after surgery and before finishing the experiment, a quick RIHP increase of at least 20 mmHg after ~ 5 s of renal vein occlusion, followed by a quick return to the baseline after renal vein disocclusion. At the end of the experiment and under deep sodium pentobarbital anaesthesia, a catheter (PE-50) was installed in the aorta through the right renal artery for anterograde perfusion of the left kidney.

### Experimental protocol

Once the carotid artery had been catheterized, a first baseline blood sample (BS1, 150 μl) was taken in pre-heparinized capillaries for measuring haematocrit (Ht) and plasma protein concentration ([Pr]p). The i.v. infusion of the maintenance solution A comprising 1% of BSA albumin (ALB, SA-Ch A-4503, Toluca, Edo de México) in 0.9% saline solution, was then started and continued throughout the surgery (57 μl min ‒1). In order to control for potential neurohumoral influences, the Maintenance Solution A was substituted, at the end of the surgery, by the Maintenance Solution B (hormonal clamp) which consisted of a solution containing aldosterone (66 ng Kg‒1 • min‒1, 0.00423 mg 10 ml‒1, Fluka 05521, SA-Ch), norepinephrine (333 ng Kg‒1 • min‒1, 0.025 mg 10 ml‒1, Pridam, Pisa, Guadalajara, Jal., México), vasopressin (0.17 ng Kg‒1 • min-1, 0.0000109 mg/10 ml, SA-Ch V9879), angiotensin II (AngII) (5 ng Kg‒1 • min‒1, 0.00032 mg 10 ml‒1, SA-Ch A9525), and an ethanol solution of hydrocortisone acetate (33 μg Kg‒1 • min‒1, 2.1 mg 10 ml‒1, SA-Ch 4126). The foregoing components were dissolved in 8 ml of Maintenance Solution A, which was i.v. infused at a rate of 57 μl/min throughout the experiment, as described by Garcia-Estañ and Roman [9]. The Roeder knots were then run to raise the renal arterial pressure (RAP) to ~ 120 or 130 mmHg, and then the RAP was controlled at 100 mmHg using a custom-made manual hydraulic micropump. Once the above has been completed, a baseline **(BL)** phase of 30 min was established, after which a second blood sample (BS2, 150 μl) was taken. In S1, undertaken approximately three later, once all variables had returned to the baseline and, according to randomization, a sudden real micro-bolus (5μl) injection of either SS + 2 mM of α-trinositol (SS + 2 α-TNS group) or SS + 4 mM of α-trinositol (SS + 4 α-TNS group) was administered into renal medullary interstitium. In S2, undertaken approximately three min later, once all variables had returned to the baseline, according to randomization, either a sham (time control group = CTR group) or a sudden real micro-bolus (5μl) injection of either solely SS (SS group) or SS + 2 mM of α-trinositol (SS + α-TNS group) was made into the renal medullary interstitium. An experimental **(EXP)** phase of 60 min was instituted, at the end of which a third blood sample was taken (BS3, 150 μl). In both series, this experimental protocol was typified as a longitudinal diagnostic sub-protocol (vide infra). Finally, under deep sodium pentobarbital anesthesia, the left kidney was fixed with a 20% buffered paraformaldehyde solution to verify the position of both the interstitial catheter and the LD probe through the coarse cross sections of the kidney.

### Analytical techniques

RAP and RIHP were recorded by coupling P23-XL transducers (Gould Inc, Oxnard, CA, USA) to a 79D Grass polygraph (Grass Instrument Co., Quincy, MA, USA) via Grass 7P1–7DA amplifiers. Both pulsatile waveform signals were electronically attenuated (0.1) and digitized at 1 Hz using a Data Translation DT 2801 analogue-digital board with HP-Vee software (Hewlett-Packard Co, Loveland, CO, USA). The RAP transducer was calibrated with a lead manometer (0–200 mmHg), and the RIHP transducer was calibrated with a high-sensitive saline manometer (0–20.4 cm saline / 0–15 mmHg). Renal outer medullary blood flow (RoMBF) recordings were obtained using a Periflux system consisting of a PF 5001 main unit, a PF 5010 LDPM (Laser class 1, 780 nm, power = 1 mV, time constant 0.3s, bandwidth = 20 Hz—20 KHz) with validated electronic linearizer [10], a probe 411, and a PF100 calibrator device. Perfusion, in arbitrary perfusion units (PU), and total backscatter signals were continuously recorded and digitized at a frequency of 1 Hz using the same HP-Vee software. The hematocrit (Ht) was measured by microhematocrit technique, using a microcapillary reader (Model 2201, International Equipment Company, Boston, MA, USA) once the sample had been centrifuged at 11,000 rpm for 10 min (Clay Adams MP centrifuge, Becton Dikinson Co, NJ, USA). The plasma protein concentration ([Pr]p) was measured by refractometry (Clinical Refractometer 5711–2021, Schuco, ERMA INC, Tokio, Japan) using the plasma derived from the microhematocrit readings. The position of the tip of the renal interstitial catheter in the three groups was always located at the boundary between the inner strip of the outer medulla and the renal papilla, while the position of the tip of the LD needle probe was always located at the boundary between both strips of outer medulla and kept separate from the interstitial catheter.

### Experimental design and definition of variables

The rats were randomly assigned via R software (V3.1) to groups when they reached a body weight between 290 and 326 g. Following the elimination criteria, the eliminated rats were substituted after the first round of experiments by others which were sequentially assigned as those were eliminated (a random process) through a spiral tracking strategy, a process which continued until the groups were completed. Because the study’s main response variable was a difference (ΔRIHP), the study was classed [11] as a two-way completely randomized cross-sectional study containing a longitudinal diagnostic sub-protocol (vide supra). The two ways were group with three levels, and time with repeated measurements throughout. The RAP was the controlled variable, Ht and [Pr]p were hemodilution indicator variables, and RIHP before, during, and after either the sham or real sudden micro-bolus (5 μl) injection into the renal medullary interstitium, was the primary measured variable. The RoMBF (PU) before, during, and after either the sham or real sudden micro-bolus injection was the secondary measured variable. Series 1 was performed with two purposes: (a) to establish an effective dose by exploring the time courses and magnitudes of both ΔRIHP and %ΔRoMBF responses to two different doses (2 and 4 mM) of α-TNS; and, (b) to defining the α-TNS dose to be used in the Series 2. These doses were established taking into account the following considerations: 1) the cell membrane is almost impermeable to the water-soluble α-TNS [12]; 2) as α-TNS renal distribution volume is unknown, we assumed that it would be distributed at least into the renal interstitial volume whose magnitude is ~ 70 μl / 100 g of bw [13]; 3) the renal interstitial volume is mainly found (96%) in the renal medulla [14]; 4) the selective application of a drug into the renal medullary interstitium localizes the drug within it by the countercurrent trapping mechanism [15]; 5) the bolus volume to be injected would be of 5 μl (micro-bolus), 6) the α-TNS Kd reported in cardiac membranes is of 160 nM [12]; 7) the effective drug concentration was estimated at three times its Kd [16], namely 500 nM or 0.5 μM, and, 8) extracellular renal catabolism rate of α-TNS is about 20% in ⁓ 6 min [17]. Thus, 0.01 μmol of α-trinositol in 5 μl (2000 μM or 2 mM) suddenly applied and dissolved in ~ 200 μl (95% of 210) of renal medullary interstitial volume would give an initial concentration of 50 μM (100 times the effective concentration). Moreover, after after 30 min, in the least favorable circumstances, such a concentration could drop to 0.5 μM (one effective concentration). The sample size (n) for S2 was calculated after performing the first twelve random experiments. Given α = 0.01, a root mean square error (σ = 1.2536), a δ of at least 1 mmHg between SS and SS + α-TNS groups (at minute 30 post-injection) and 80% power, the least significant number was nine experiments for each of the three groups. This δ value was chosen because the reported change in interstitial hydrostatic pressure induced by α-TNS in rat skin [6] corresponds to that order of magnitude. The renal interstitial hydrostatic pressure difference (ΔRIHP) is equal to EXP RIHP minus the average BL RIHP, and the %ΔRoMBF is the % change of RoMBF taken as 100% the average of BL PU. With the exception of hematocrit (Ht) and plasma protein concentration ([Pr]p), the remaining variables were measured continuously.

### Statistical analysis

The overall time course of each variable (Ht, [Pr]p, RAP, RIHP, ΔRIHP and %ΔRoMBF) in each series was analysed (95% CI) using two way repeated measures MANOVA (2WRM-MANOVA) [18], one way being group and another being time with repeated measurements, before, during, and after a sudden micro-bolus injection looking for time, group, and/or time * group interaction effects. To assess the effect of time on each group, a RM-MANOVA by group was performed. In each experiment, the standard error of the mean at zero time point taken for this analysis was the standard error of the difference between the average of the 30 min BL RIHP time point minus the average of the 26 min BL RIHP time point. The response variables were modelled by linear fixed models (regression analysis, 1WANOVA), which were subject to Box-Cox transformation when they did not fulfill the parametric statistical criteria [19]. To minimize the correlation between the mean and variance over time, the %ΔRoMBF raw data underwent log 10 transformation. The best model was chosen on the basis of the highest r^2^ (determination coefficient = explained variation), the lowest Akaike Information Criterion (AIC) [20], and the parsimony principle. Post-hoc multiple comparisons were performed through a Tukey test, and the model’s residuals were tested for normality (Shapiro Wilk test) and homoscedasticity (Brown-Forsythe test) [21]. The alpha level imposed was 0.05. Statistical analysis was performed using JMP V10 (SAS Institute, Cary, NC, USA) software and all values are mean ± standard error of the mean.

## Results

### Series 1

**Fig 1:**
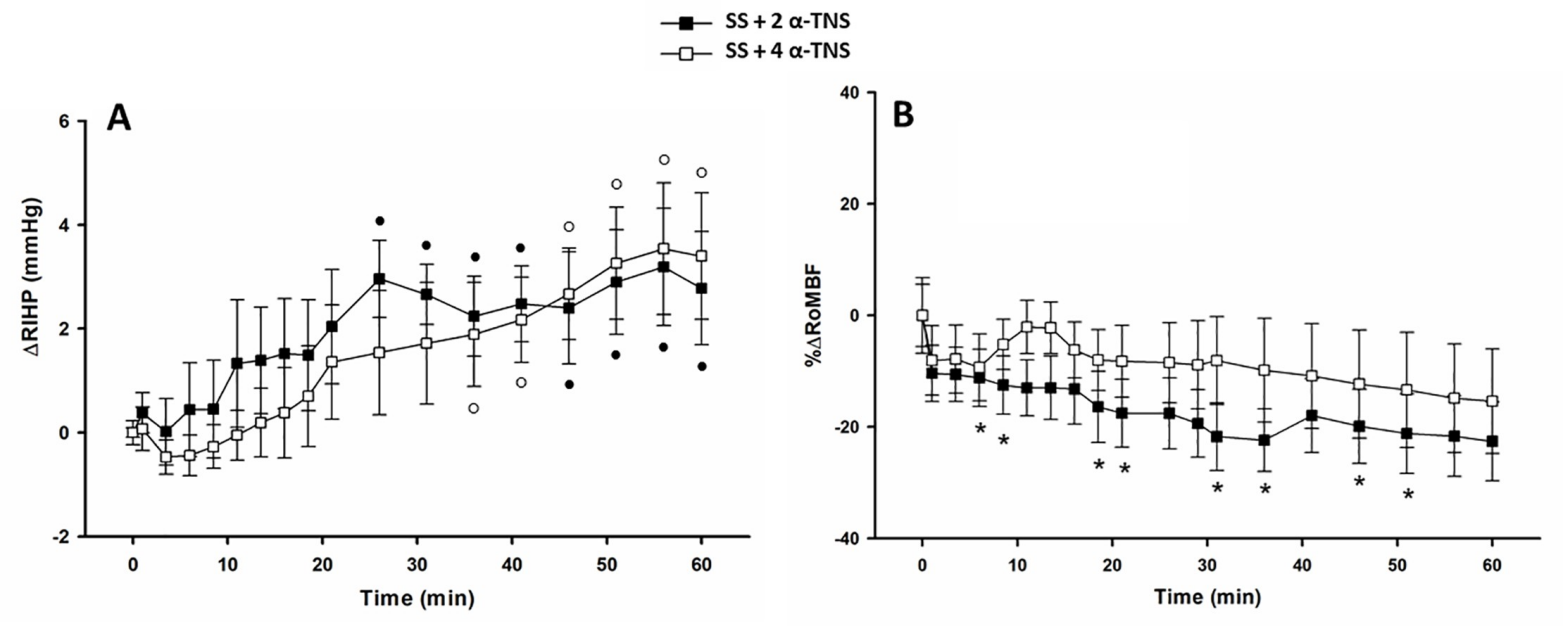
**Time courses for ΔRIHP (A) and %ΔRoMBF (B) in S1.** In A and B, each symbol represents the delta average (A) or the %ΔRoMBF (B) of the last 30 sec of each 2.5 min that elapsed during the first 20 min and of each 5 min that elapsed during the next 40 min after the sudden bolus injection (EXP phase) taken as BL value the average of RIHP (A) or RoMBF (B) recorded during the 30 min that the BL phase lasted. The overall RM-MANOVA indicated time effect (P = 0.0025 and P = 0.045) for ΔRIHP and %Δ RoMBF respectively, but not group nor time * group interaction effect. RM-MANOVA by group for ΔRIHP: ● P from 0.03 to 0.02 vs. 0T in SS + 2 α-TNS group (n = 4), ○ P from 0.04 to 0.02 vs. 0T in SS + 4 α-TNS group (n = 5). RM-MANOVA by group for %ΔRoMBF: * P from 0.03 to 0.0032 vs. 0T only in SS + 2 α-TNS group.

The baseline (BL) values of renal interstitial hydrostatic pressure (RIHP) were 3.7 ± 0.77 mmHg (n = 4) and 3.2 ± 0.54 mmHg (n = 5) for SS + 2 α-TNS and SS + 4 α-TNS groups, respectively. Fig 1 shows in panel (A) the time course of RIHP difference (ΔRIHP) and in panel (B) the time course of % change of renal outer medullary blood flow (%ΔRoMBF) in response to the sudden 5 μl bolus injection of SS + 2 mM α-TNS and SS + 4 mM α-TNS into renal medullary interstitium. While the overall analysis indicated a time effect for both ΔRIHP (P = 0.0025) and %ΔRoMBF (P = 0.045), no group or time * group interaction effects were indentified for either of the two variables. The SS + 2 α-TNS group presented a progressive increase in ΔRIHP that reached a significant (P = 0.05) and maximum value (3.0 ± 0.74 mmHg vs. 0 min time point) at the 28 min time point, and then, with slight variations, remained at that level for the next 30 min. This response was accompanied by a slow but progressive and significant decrease (~ 21%) in %ΔRoMBF. In contrast, the SS + 4 α-TNS group (n = 5) showed a progressive but slower increase in ΔRIHP which was significant (P = 0.02) from the 36 min time point onwards, and which reached its maximum value (3.5 ± 1.3 mmHg) at the 56 min time point. Such a response was not accompanied by any significant decrease in %ΔRoMBF with respect to BL value. Thus, although the maximal increase of ΔRIHP was similar with both α-TNS doses, the peak value was reached faster in the SS + 2 α-TNS group than in the SS + 4 α-TNS group and once the peak ΔRIHP value was reached, it remained at that level for the rest of the experiment. Furthermore, less data scattering was found in both ΔRIHP and %ΔRoMBF data in the SS + 2 α-TNS group.

### Series 2

#### Final body weight and experimental evolution of renal arterial pressure (RAP)

**Fig 2:**
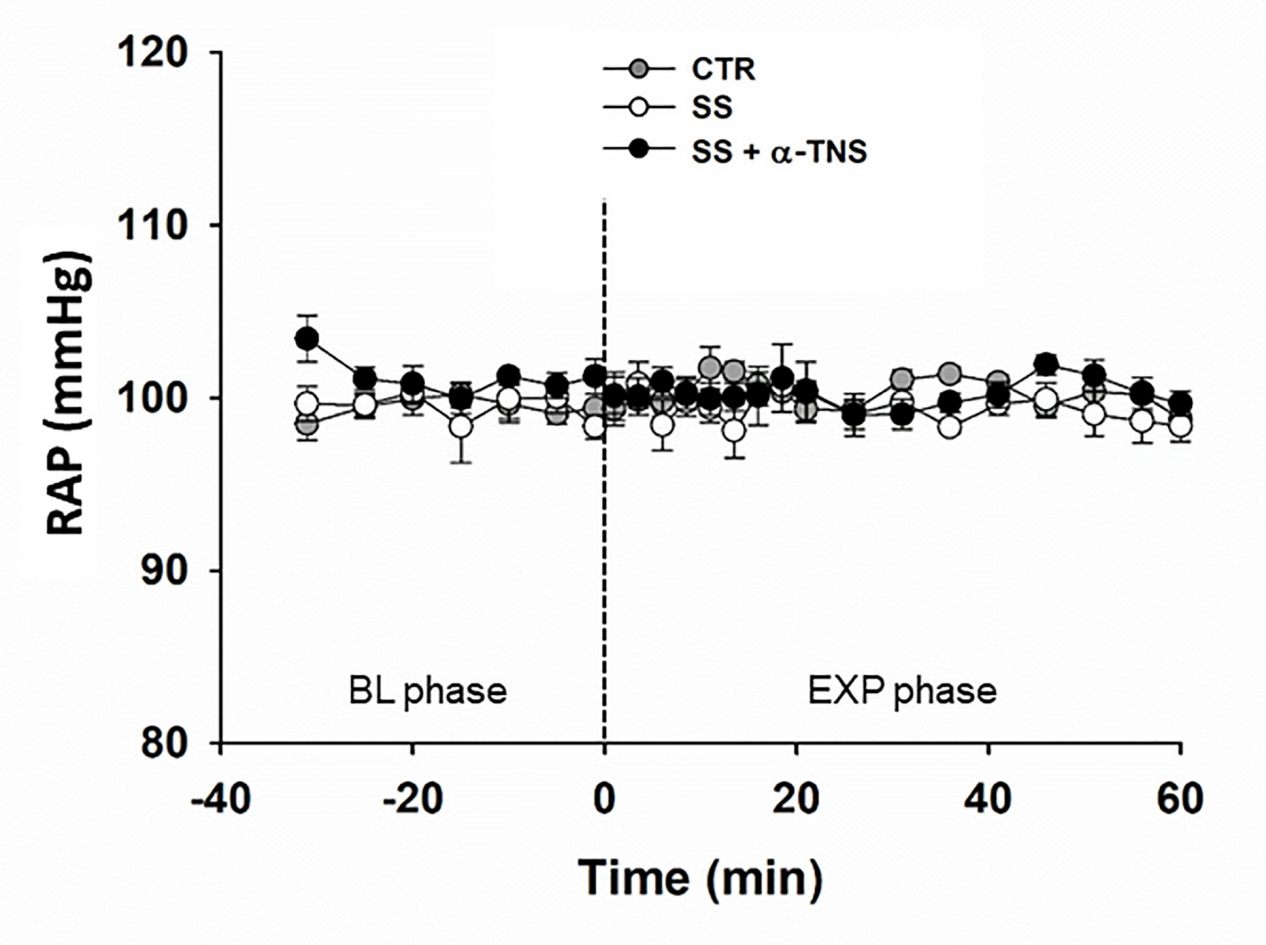
Time course of RAP in Series 2. CTR group: n = 9, SS group: n = 9, SS + α-TNS group: n = 9. The symbols represent the average of the last minute of each 5-min recording during the BL phase and the average of the last minute of each 2.5-min recording over the 20 min and the average of each 5-min recording over the next 40 min of the EXP phase. The overall RM-MANOVA indicates no time, group or time*group interaction effects.

The final body weight in the CTR (n = 9), SS (n = 9), and SS + α-TNS (n = 9) groups was 305 ± 2, 314 ± 1, and 307 ± 3 g, respectively. The evolution of the RAP throughout the experiment in the three groups is shown in Fig 2. To assess the quality of RAP control at 100 mmHg, the RAP time course was analysed using the average of the last 60 s of recording for each time point (7 at the BL phase and 17 at the EXP phase). The overall analysis indicated that there were no time, group, or time * group interaction effects, demonstrating a good experimental control of RAP at 100 mmHg.

#### Time course for haematocrit (Ht) and plasma protein concentration ([Pr]p)

**Fig 3:**
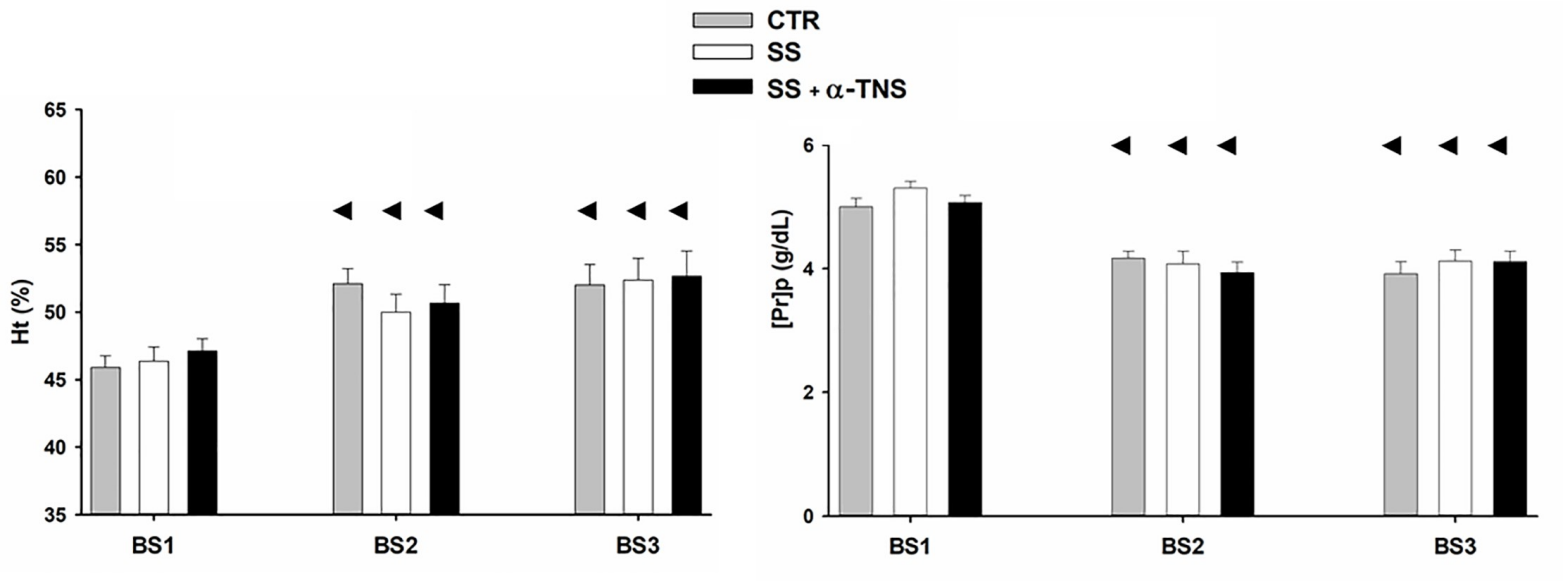
Time course for Ht and [Pr]p in S2. CTR group: n = 9, SS group: n = 9, SS + α-TNS group: n = 9. The blood samples were taken at the beginning of the surgery (BS1), prior to performing the sudden bolus injection (BS2), and at the end of the experiment (BS3). The overall RM-MANOVA showed only time effect (P < 0.0001) for both variables. ◄ P < 0.0001 vs. BS1 by group RM-MANOVA.

The time courses for Ht and [Pr]p are shown in Fig 3. The initial Ht and [Pr]p measured at BS1 did not show differences among groups. The overall analysis indicated only time effect (P < 0.0001). Compared to BS1 values, the Ht had increased significantly (P < 0.0001) by ~ 10% in all groups by BS2, whereas the [Pr]p decreased significantly (P < 0.0001) by ~21% in all groups by BS2. By the end of the experiment (BS3), 60 min after the sham or real sudden bolus injection, the Ht and [Pr]p values showed no change compared to the BS2 values in any of the groups.

#### Baseline RIHP values and the time course for ΔRIHP and %ΔRoMBF

**Fig 4:**
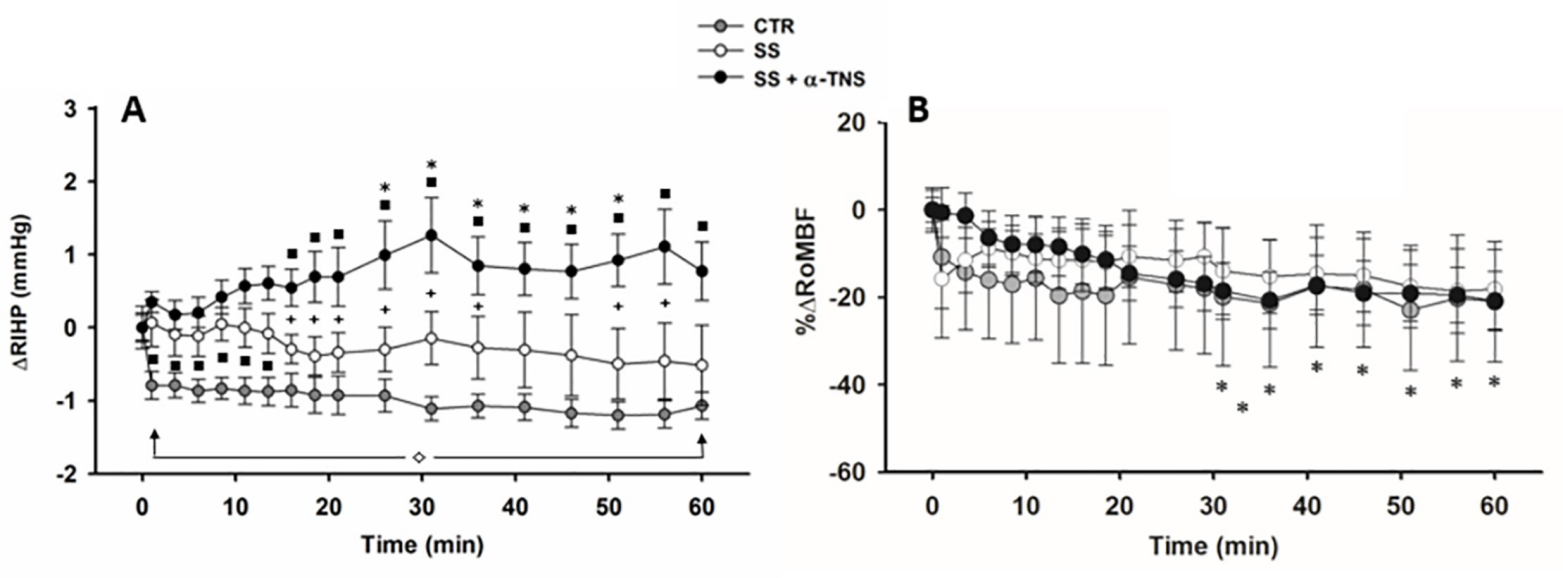
**Time courses for ΔRIHP (A) and %ΔRoMBF (B) in S2.** CTR group: n = 9, SS group: n = 9, SS + α-TNS group: n = 9. In A and B, each symbol represents the same as in the Fig 1. The overall RM-MANOVA conducted for ΔRIHP indicated time*group interaction (P < 0.0177) and group (P = 0.0004) effects but no time effect. RM-MANOVA by group for ΔRIHP (1 tailed): ◊ P from 0.0023 to 0.0001 vs. 0T in CTR group; * P from 0.05 to 0.025 vs. 0T in SS + α-TNS group; 1WANOVA + Tukey test (1 tailed): **+** P from 0.04 to 0.017 in SS + α-TNS group vs. SS group; ■ P from 0.03 to 0.008 in SS group vs. CTR group or from 0.007 to 0.0002 in SS + α-TNS group vs. CTR group. At the remaining time points there was no difference in ΔRIHP between SS and CTR groups. The overall RM-MANOVA conducted for %ΔRoMBF found only a time effect (P = 0.0012). The RM-MANOVA by group for %ΔRoMBF (1 tailed): * P from 0.03 to 0.01 vs 0T only in the SS + α-TNS group.

The baseline (BL) values for RIHP were 3.0 ± 0.34, 3.1 ± 0.49, and 3.2 ± 0.30 mmHg for the CTR, SS, and SS + α-TNS groups, respectively. The time course for ΔRIHP is shown in Fig 4A. The overall analysis revealed time * group interaction effect (P < 0.018), and group effect (P < 0.0004), but no time effect. The SS + α-TNS group (2 mM) showed a slow and progressive ΔRIHP increase which can be differentiated from the CTR group from the first minute onwards, and is differentiable from the SS group in two lapses occurring from minute 16 to minute 36 and from minute 51 to minute 56. This ΔRIHP response reached its maximum value (1.3 ± 0.5 mmHg) compared to the zero time point at 31 min (P = 0.025) and then, with slight variations, remained at that level for the next 25 min. This size of the effect of the ΔRIHP at 31 min was similar to that reported in rat skin [6] and constituted a ~100 and ~200% higher response than that was observed for the SS and CTR groups, respectively. The time course for %ΔRoMBF is shown in Fig 4B. The overall %ΔRoMBF analysis showed only a time effect (P = 0.0012), which primarily depended on the %ΔRoMBF time course showed by the SS + α-TNS group (P from 0.03 to 0.01), which decreased by ~21%. However, no statistically significant differences were found among the groups at any time point. A correlation analysis was conducted between the ΔRIHP and %ΔRoMBF at each time point for the three groups in order to identify the possible association between them, finding no such association between these two variables at any time point for any of the three groups.

## Discussion

The most significant new findings from this study performed on hydropenic rats, are as follows: (a) The sudden micro-bolus (5 μl) injection of solely SS into the renal medullary interstitium is unable to increase the ΔRIHP; (b) The non-injection of a micro-bolus of SS (sham, CTR group) into the renal medullary interstitium is accompanied by a decrease in ΔRIHP; and, (c) A sudden micro-bolus (5 μl) injection of SS + 2 mM α-Trinositol (α-TNS) into the renal medullary interstitium is accompanied by a differential increase in ΔRIHP that is ⁓ 200% higher than that found for the CTR group and ⁓ 100% higher than that found for the SS group. These responses were observed under conditions of acute renal denervation, hormonal clamp, and RAP control at 100 mmHg, and were not associated with differential changes among the three groups in the %ΔRoMBF or hemodilution parameters (Ht and [Pr]p). As far as we could ascertain, this study has no precedent in the literature and represents both the first time that the acute effects of local tissue, as opposed to i.v., administration of α-TNS have been reported and the first time they have been tested in renal tissue.

Alfa-trinositol (α-TNS) was used in the present study because it has well-proven anti-inflammatory effects in several models of inflammation [5,6,7], significantly and markedly reducing both the typical increased negativity of interstitial pressure [5,6,7] and the albumin extravasation commonly found in these models [5]. Therefore, it has an edema reducing effect. Although its intimate mechanism of action is unknown, all the evidence points to it resulting from the centripetal stretching of the fibrillary collagen network that surrounds the extracellular matrix (prone to swelling due to its hyaluronic acid content) as a consequence of the induction of fibroblastic contraction [6]. The latter appears to be mediated by α_2_β_1_ integrins [6] as well as by an increase in fibroblast intracellular Ca^++^, which, apparently occurs independent to both PI3-kinase and IP3 [22]. The next step in the present study was to determine the ideal volume of the micro-bolus to be injected and the effective dose of α-TNS for a single local renal injection. To avoid inducing significant rmDIVE via a 100 μl bolus infusion of SS, which by itself increases RIHP [4], preliminary experiments suggested the use of a sudden 3–5 μl micro-bolus injection. We exploited the countercurrent drug-trapping mechanism present in the renal medullary interstitium, which enables drugs to be retained longer with very small quantities escaping into systemic circulation [15]. Considering the additional six pharmacokinetic elements described in the Material and Methods section of the present study, we decided to conduct preliminary testing on the effectiveness of two doses of α-trinositol (2 and 4 mM) integrated into the micro-boluses of SS (Series 1, SS + 2 mM and SS + 4 mM groups). On the basis of the positive association between BL RIHP and EXP RIHP [4] and the similarity of BL RIHP values among the groups, we focused more on the time course for ΔRIHP than the time course for absolute RIHP. A similar maximal increase of ΔRIHP and a similar maximal decrease of %ΔRoMBF were found with both α-TNS doses, with no overall differences observed in the time courses for ΔRIHP and %ΔRoMBF between groups. However, the SS + 2 α-TNS group reached the ΔRIHP peak faster and remained at that level for the rest of the experiment. Furthermore, as there was also less scattering in both the ΔRIHP and %ΔRoMBF data, a 2 mM of α-TNS dose was chosen for Series 2.

Series 2 was designed to test whether the sudden micro-bolus injection of SS + 2 mM of α-TNS into the renal medullary interstitium was able to induce a contraction of the renal interstitium, as indicated by a differential increase in ΔRIHP. The similar BL RIHP values found for the three groups (CTR, SS and SS + α-TNS) in this series indicate that the randomization of the experimental units to the groups was adequately carried out. The present study focused more on measuring ΔRIHP more than absolute RIHP because, in addition to the rationale described above, we wanted identify the size effect induced by α-TNS with greater precision. A similar body weight among groups and a similar increase in HT and decrease in [Pr]p values immediately prior to the micro-bolus injection (BS2), in all the groups, indicates that the body hydration status (hydropenia) with which the animals (and the kidneys) faced the experimental maneuver (either sham or real sudden micro-bolus injections) was similar. The divergent behaviour of Ht and [Pr]p at BS2 could be explained by an increase in protein permeability produced by the i.p. administration of sodium pentobarbital on peritoneal capillaries [23]. Because the time courses for Ht and [Pr]p were equal for the three groups of this series, it is believed that these two variables had no influence on the evolution of the ΔRIHP response to either the sham or real micro-bolus injection throughout the experiment.

As expected, given the findings of the preliminary experiments (see Material and Methods), we observed that the sudden micro-bolus (5 μL) injection of solely SS into the renal medullary interstitium was unable to increase ΔRIHP over time (SS group). On the other hand, in the group in which there was not sudden micro-bolus injection into such a space (CTR group) ΔRIHP notably and significantly decreased by ⁓ 1 mmHg over time. These findings indicates that: (a) under the experimental conditions described here and by the time at which the sudden injection started, a SS micro-bolus of at least 5 μl was either unable to modify the ΔRIHP or was sufficient to maintain it without change; and (b) an interstitial volume deficit of just 5 μl (~2.5% of the renal interstitial volume) causes a decrease in ΔRIHP, which suggests that the renal medullary interstitium may function as a detector of interstitial volume.

Alfa-trinositol (α-TNS) has been shown to stimulate the contraction of fibroblast *in vitro* [6] and *in vivo* [5, 6, 7] although by mechanisms not yet well identify (vide supra). The fact that the sudden micro-bolus injection of SS + 2 mM of α-TNS into the renal medullary interstitium produces a gradual increase in ΔRIHP, while the micro-bolus of solely SS does not, demonstrates that this drug acts on the renal medullary interstitium and strongly suggests that this effect is achieved by activating the renal fibroblast contractile machinery, although alternative explanations would need to be excluded (vide infra). Additional supporting evidence in favour of the activation of contractile machinery of the renal fibroblast can be found by contrasting the time courses of the ΔRIHP responses in the three groups. So, the ΔRIHP response found in the SS + α-TNS group was: (a) undifferentiable from the response shown by the SS group during the first 13 min post-injection, (b) significantly different with respect to the zero min time point from 26 min time point onwards, (c) significantly different with respect to the response showed by the CTR group from the first minute time point onwards, and (d) significantly different with respect to the response showed by the SS group from 16 min time point and during the following 20 minutes (up to 36 min time point), and then from 51 min to 56 min time point. Together, all this indicates that while the irrelevant RIHP effect of the solely SS micro-bolus occurs and the drug spreads through the renal interstitium, the latter begins to manifest a reactive response to the drug ~ 15 min after its sudden injection. The overall ΔRIHP evolution in the SS + α-TNS group is coincident with the evolution of the attenuated decrease of interstitial fluid hydrostatic pressure observed by Lund et al. [5] in thermally injured rat skin when α-TNS was i.v. administered 5 min after inducing the injury.

There are several alternative but unlikely explanations for the ΔRIHP increase observed in the SS + α-TNS group. One of them would be that the renal medullary blood flow decreased differentially (vs. CTR and SS groups), causing a much lower output of liquid from the renal medullary interstitium with the consequent increase in ΔRIHP. Although the experimental design controlled most of the potential sources of variation, the high variability in the %RoMBF values could have masked small differences among the groups in both series. The generalized similar (~20%) decrease in %ΔRoMBF observed in all Series 2 groups, along with the absence of a clearly divergent behaviour among groups, leads to us to conclude that such a generalized RoMBF decrease is a reflection of the hydropenic status that all the rats experienced, which was manifested in the tendency of the Ht to be maintained or increased, when the BS3 sample was taken (vs. BS2). Evidence in rats [24] indicates that i.v. infusion of high doses (80 mg Kg‒1 • h‒1) of α-TNS more often produces an increase in total renal blood flow, than a decrease. However, it should be recognized that the effects of local intravascular and extravascular application of α-TNS on the vasa recta or renal arterioles have not been reported. Interestingly, the ΔRIHP in the SS + 4 mM α-TNS group from Series 1 tended to increase more slowly and the %ΔRoMBF tended to decrease more slowly than those observed in the SS + 2 α-TNS group. All this would suggest: (a) that the dose of 4 mM of α-TNS, in contrast to the 2 mM dose, was able to induce some renal medullary vasodilation, (b) that such medullary vasodilation may have caused a greater outflow of liquid from the renal medullary interstitium, which, together with the activation of the fibroblast contractile machinery may have exacerbated the reduction (by squeezing) of the renal interstitial volume, which may, in turn, explain the slower increase in ΔRIHP in the SS + 4 mM group of Series 1. The absence of a differential response in %ΔRoMBF among groups in the Series 2 and the fact that we did not find any correlation between ΔRIHP and %ΔRoMBF, at any time point for any of the three groups, supports the idea that the ΔRIHP changes observed in Series 2 were not related a changes in renal medullary blood flow. So and then, the effect of 2 mM α-TNS dose on ΔRIHP in both series can more probably be explained the sole activation of fibroblast contractile machinery (without renal medullary vasodilation) with the consequent squeezing of the interstitial matrix, though in minor extent that with 4 mM dose, and therefore with a relatively higher renal medullary interstitial volume (vs. 4 mM dose).

Other alternative but unlikely explanations for the increase in ΔRIHP in the SS + α-TNS group of Series 2 may be: (a) That this drug decreases urinary sodium and water excretion. Experiments in rats [25] where α-TNS had been infused i.v. demonstrated that this drug propitiated the opposite, that is to say, natriuresis and diuresis. In addition, Khraibi et al. [26] demonstrated that significant natriuresis and diuresis induced by i.v. furosemide did not have an effect on RIHP values, at least in euvolemic rats; (b) That α-TNS increases the renal medullary capillary protein permeability, although Lund et al. [5] reported, in a thermally injured skin model, that i.v. α-TNS decreases albumin extravasation in injured skin but does not alter the albumin extravasation in non-injured skin; (c) That α-TNS increases rapidly the hyaluronic acid (HA) content of the renal medullary interstitium. So far, there is no evidence that α-TNS can increase or decrease renal HA content, and Stridh et al. [27] have documented that the HA renal turnover is relatively slow; (d) That α-TNS induces the osmotic movement of water into the interstitium with the consequent increase in ΔRIHP, also bearing in mind that the renal medulla concentrates solutes. Taking into account the fact that the cell membrane is almost impermeable to the water-soluble α-TNS [12], the osmotic contribution of the doses of α-TNS used (2 and 4 mM) would be only of ~ 0.01 μosm or 0.02 μosm respectively, in the micro-bolus (5 μl) of SS injected. Taking an average renal interstitial osmolality of 750 mOSM, the osmotic content in the ~200 μl of renal medullary interstitial volume [13] would be 150 μosm, meaning that the renal medullary osmotic content pots-injection would correspond to either 150.01 or 150.02 μosm in the new renal medullary interstitial volume of 205 μl. Over time, these values would not substantially change along the entire cortico-medullar gradient installed by the state of hydropenia, because the α-TNS would be substituted by its renal extracellular metabolite Ins (1,2)P2 [17]. Thus, it is unlikely that changes in the renal interstitial osmolality explain the ΔRIHP observed; and finally, (e) That α-TNS induces a decrease in renal lymphatic flow with the consequent increase in ΔRIHP, a mechanism which, although possible [28] seems unlikely as it would be contrary to the well-proven anti-inflammatory effect of the drug. It remains to be discussed, as alternative explanation, if α-TNS by increasing the frequency and or amplitude of renal pelvic wall peristaltic contractions [29] could increase the RIHP. As far as we know, the effect of renal pelvic wall peristaltic contractions on RIHP have not been reported, so that to define the feasibility of this alternative further research on the effect of α-TNS on the renal pelvic wall peristalsis and urological smooth muscle cell contraction would be needed.

## Conclusions

In conclusion, the results obtained in Series 1 and 2 under conditions of no rmDIVE and judged in light of the existing evidence to date, indicate that the injection of α-TNS into the renal medullary interstitium induced the contraction of the renal interstitium *in vivo*. This reinforces the feasibility of our preliminary conclusions [4] and if confirmed the renal tissue would be the third tissue, the other two being the pulmonary alveolar septa [30] and the cellular subcutaneous tissue [2]), for which there is either direct or indirect evidence that their interstices contracts *in vivo*. It is worth noting that the present study was not designed to assess how an increase in ΔRIHP, induced in this case by α-TNS, would affect either the tubular function or renal medullary interstitial dynamics. The potential physiological implications that the renal interstitium contracts *in vivo* could be multiple. One of them would be that it participates in the process of urinary concentration, as the antidiuretic hormone (ADH) has been shown to be capable of: (a) contracting the renal medullary fibroblasts *in vitro* [3], and, (b) modifying the turnover of renal hyaluronic acid and aquaporins [27]. Another physiological implication would be that the control of the renal interstitial contraction by renal autacoids (e.g. endothelin [3], 20-HETE [31]) may also modulate the complex and still not well understood interactions between PHIR and the tubular sodium transport [31]. A third physiological or pathophysiological implication would be that, by controlling mechanics of the extracellular matrix (particularly its stiffness), the renal interstitial contraction may be involved in the stem cell differentiation, an event underlying the phenomenon of tissue fibrosis [32]. It is possible that the fibroblast-mediated contraction of the renal interstitium along with the compression of the renal interstitium induced by peristaltic contraction of the renal pelvis [33] may be integrally involved in the above implications.
